# Primary mesenchymal chondrosarcoma of the kidney with synchronous implant and infiltrating urothelial carcinoma of the ureter

**DOI:** 10.1186/1746-1596-7-125

**Published:** 2012-09-21

**Authors:** Hua Xu, MuMin Shao, HuiLi Sun, ShunMin Li

**Affiliations:** 1 Departments of Pathology, Shenzhen Affiliated Hospital, Guangzhou University of Traditional Chinese Medicine, Shenzhen, China; 2 Departments of Nephrology, Shenzhen Affiliated Hospital, Guangzhou University of Traditional Chinese Medicine, Shenzhen, China

**Keywords:** Mesenchymal chondrosarcoma, Urothelial carcinoma, Kidney, Ureter

## Abstract

Primary mesenchymal chondrosarcoma of the kidney is rare, and it shows distinct undifferentiated tumor cells and well differentiated cartilagenous components. Also assident infiltrating urothelial carcinoma of the ureter is an extremely rare cancer. We report a case of primary mesenchymal chondrosarcoma occurring in the left kidney with an ipsilateral and distinct distal ureteric implant, and a coexisting infiltrating urothelial carcinoma of the ureter in a 64-year-old man. Histopathological examination and immunohistochemical studuies showed the classic features of mesenchymal chondrosarcoma in kidney, as well as a few infiltrating urothelial in ureter. Multitarget fluorescence in situ hybridization (FISH) suggested that the development of the urothelial carcinoma in the ureter may be triggered or induced by the chondrosarcoma component. The patient died 2 month after left nephro-ureterectomy. This is the first reported case of a primary mesenchymal chondrosarcoma of the kidney with coexisting infiltrating urothelial carcinoma of the ureter.

**Virtual Slides:**

The virtual slide(s) for this article can be found here: http://www.diagnosticpathology.diagnomx.eu/vs/1522835667751019

## Background

Mesenchymal chondrosarcoma was first reported by Lichtenstein and Bernstein in 1959 [1]. This malignant tumor mostly arises in the skeleton, and one third of the cases arise in the soft tissue and other organs. Extraskeletal mesenchymal chondrosarcoma is rare, and uaually arises in the head and neck region, followed by the lower extremity, the trunk and the retroperitoneum. Primary mesenchymal chondrosarcoma of the kidney is extremely rare, and the first case was published 25 years ago by Malhotra [2]. To date, there are only seven cases of primary renal chondrosarcoma reported in the English literature [2-7]. We report another case of mesenchymal chondrosarcoma of the kidney with synchronous implant and a coexistent infiltrating urothelial carcinoma of the ureter firstly.

## Case presentation

A 64-year-old man presented with gross hematuria and vague pain in the left loin. His medical history was unremarkable. Physical examination revealed percussion pain over the left kidney region. Urinalysis showed positive for protein and red blood cells. Abdominal B ultrasonography revealed left hydronephrosis and a hypoechoic mass in the inferior segment of the left ureter. Magnetic resonance imaging (MRI) confirmed left hydronephrosis, a low signal mass in the upper pole of the kidney, and another mass with high T1, low T2 signal in the inferior segment of the left ureter. Imaging examination showed no abnormality elsewhere. A clinical diagnosis of malignancy of the left kidney and the ureter was made, for which a left nephro-ureterectomy was performed.

**Figure 1:**
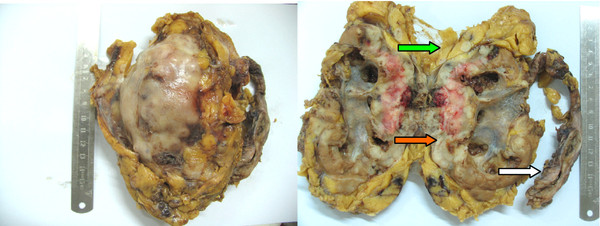
**Nephro-ureterectomy specimen showing the large tumor nodule in the kidney.** Gross examination showed the tumour involved the whole kidney cortex, medulla, capsule (red arrow) and adeps renis (green arrow) and the ureter was distended by a narrow solid tumor mass (white arrow).

**Figure 2:**
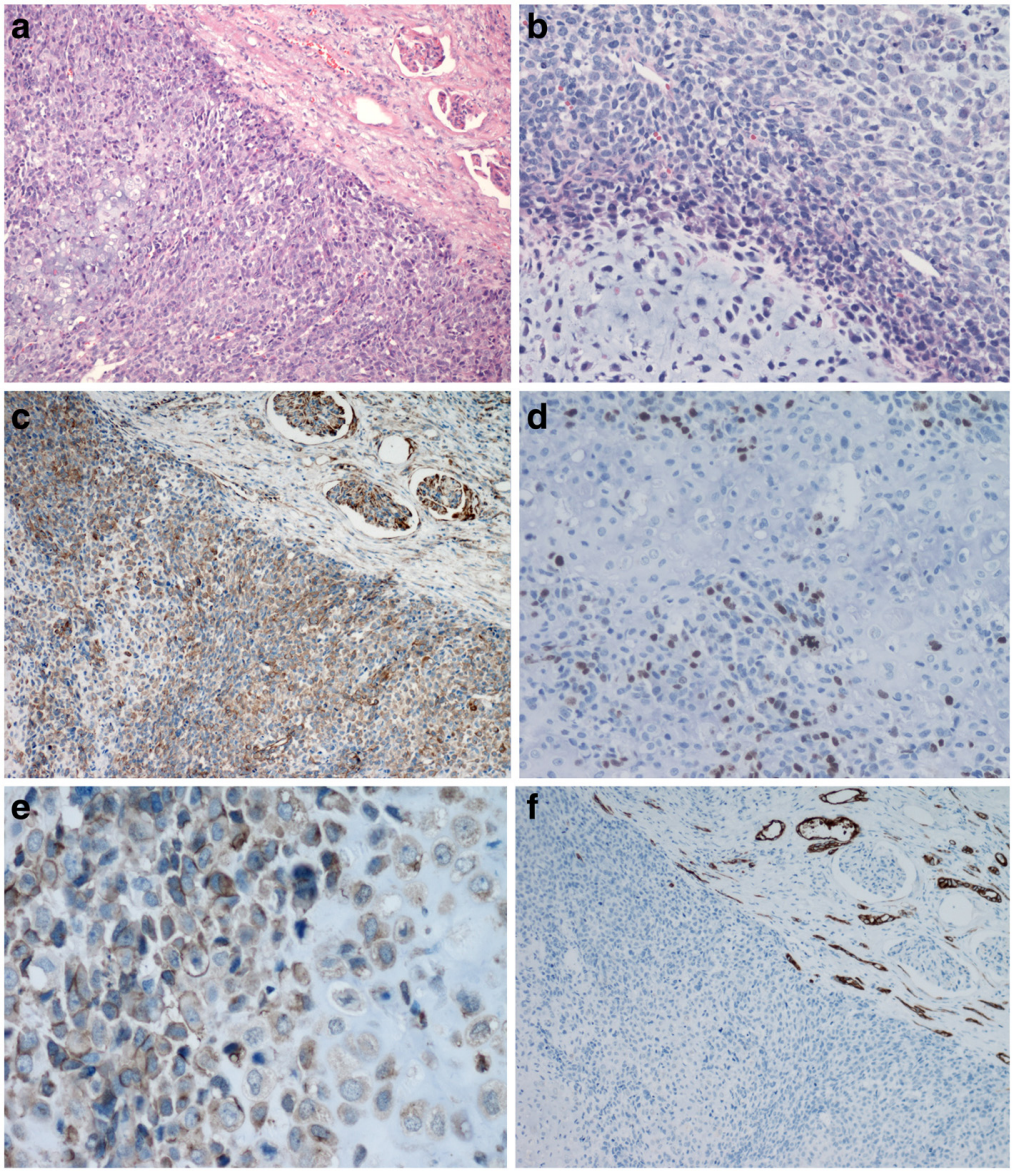
**The renal mass consisting of sheets of undifferentiated mesenchymal cells (H&E, 2a), and scattered well demarcated islands of differentiated cartilage (H&E, 2b).** Both the undifferentiated tumor cells and cartilaginous islands were immunoreactive to Vimentin (2**c**), to S-100 protein (2**d**) and to CD99 (2**e**), and not to cytokeratin (2**f**).

Gross examination of the nephro-ureterectomy specimen showed pale grayish solid tumor in the enlarged kidney, and the tumor measured 11 × 8 × 6 cm, causing dilatation of the renal pelvis. The tumor invaded the cortex, medulla, adeps renis and involved 90% of the total kidney, resulting in distortion (Figure 1a). And a separate elongated solid tumor mass, 7 × 1.2 cm in size, was present in the inferior segment of the left ureter, being distinct from the tumor in the renal (Figure 1b). Microscopically, the renal mass showed a biphasic infiltrative growth pattern with the typic characteristic of mesenchymal chondrosarcoma. It consisted of undifferentiated spindle to oval shaped cells, with hyperchromatic nuclei and scanty cytoplasm mainly arranged in Ewing’s sarcoma-like, lamellar or hemangiopericytoma-like patterns. Other areas demonstrated well defined islands of cartilage. The transformation from the undifferentiated cells to the cartilage cell was abrupt and interlaced(Figure 2a, 2b). The tumor had invaded the renal pelvis, but there were no evidence of papillary lesion or underlying urothelial carcinoma.

**Figure 3:**
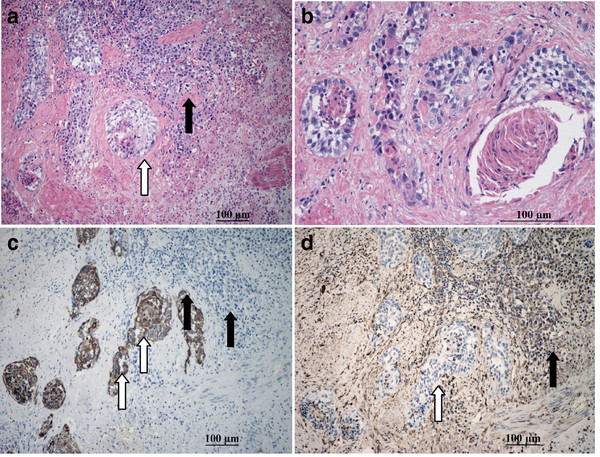
**The ureteric tumor revealing a mesenchymal chondrosarcoma, similar to that in the kidney (black arrow), and in addition a synchronous infiltrative urothelial carcinoma (white arrow) (3a),with foci of squamous differentiation and keratinization(3b).** The urothelial carcinoma (white arrow) was positive for cytokeratin (3**c**) and negative for Vimentin (3**d**); while the mesenchymal chondrosarcoma (black arrow) was negative for cytokeratin (3**c**) and positive for Vimentin (3**d**).

The tumor in the distal ureter was microscopically similar to that in the kidney, with the typical biphasic mesenchymal chondrosarcoma component , which constituted bulk of the tumor mass. However, there was a small component exhibiting features of urothelial carcinoma. The urothelial carcinoma showed infiltrative nests, of moderate cytological grade, and displayed foci of squamous cell differentiation with some degree of keratinization (Figure 3a, 3b). The mucosa adjacent to the tumor showed no evidence of underlying dysplasis or carcinoma in-situ. Extensive sampling of the kidney tumor did not identify a carcinomatous component. Vascular invasion of the chondrosarcoma was detected , but no carcinomous metastasis was detected.

**Figure 4:**
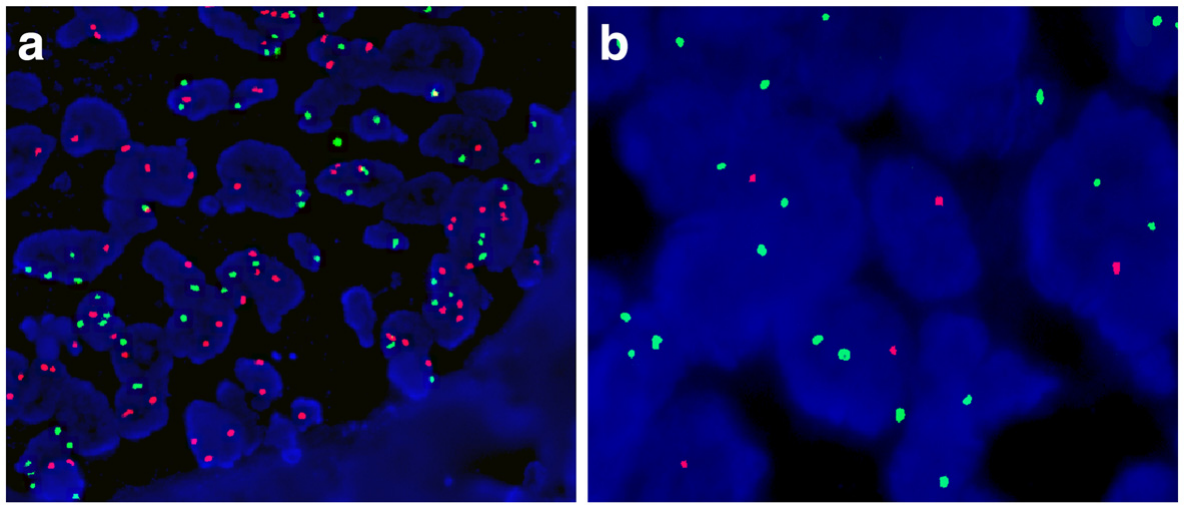
**Aneuploidy of chromosome 3(green) and 7(red) in urothelial carcinoma (**4**a**). Aneuploidy of chromosome17(green)and loss of the p16 gene(red) in urothelial carcinoma (4**b**).

Immunohistochemical staining showed the poor-differentiated tumor cells and cells within the cartilaginous areas in both the kidney (Figure 2c, 2d, 2e, 2f) and the ureter were positive for Vimentin, CD99and S-100 protein but were negative for cytokeratin, epithelial membrane antigen ,E-cadherin, Leu7, neuron-specific enolase, SMA, CD34, p63 and desmin. The poor-differentiated tumor cells displayed a higher Ki-67 index than those in the well-differentiated cartilaginous areas. The urothelial carcinoma component within the ureter, the tumor cells were positive for cytokeratin (Figure 3c), epithelial membrane antigen, E-Cadherin and negative for Vimentin (Figure 3d), S-100 and CD99, enhancing the contrast of synchronous urothelial carcinoma from mesenchymal chondrosarcoma. For FISH analysis, labeled probes specific for chromosomes 3, 7, 17 and for the p16 (9p21) gene (GP Medical Technologies,Ltd, Beijing, China) were used. Two DNA- probes were mixed together as a set double-target FISH and paired as follows: chromosome 3 (fluorescein isothiocyanate) and chromosome 7 (rhodamine), chromosome 17 (fluorescein isothiocyanate) and p16 (rhodamine).In both the urothelial carcinoma and mesenchymal chondrosarcoma components, aneuploidy of chromosome 3, 7 and 17 and loss of p16 gene were observed (Figure 4, and Tables 1, 2).

**Table 1:** The ratio of gene copy numbers to chromosome 3,7and 17 centromere by FISH probes in urothelial carcinoma and mesenchymal chondrosarcoma

**Urothelial carcinoma**				**Mesenchymal chondrosarcoma**		
chromosome	3	7	17	3	7	17
monosomy(%)	8	15	8	12	13	15
disomy(%)	35	42	29	51	61	46
trisomy(%)	27	23	40	18	16	28
polysomy(%)	30	20	23	19	10	11
aneuploidy(%)	65⋆	58▵	71◇	49⋆	39▵	54◇

Relationship between aneuploidy of chromosome 3,7, 17 of urothelial carcinoma and mesenchymal chondrosarcoma. ⋆*x*^2^ = 5.22,p = 0.022. ▵*x*^2^ = 7.23,p = 0.007. ◇*x*^2^ = 6.17,p = 0.013.

**Table 2:** Gene copy numbers of the p16 (9p21) gene by FISH probe

**Urothelial carcinoma(%)**		**Mesenchymal chondrosarcoma(%)**
single signal	36	46
double signals	39	39
three signals	0	0
no signal	25	15
gene loss	61	61

The ratio of the loss of p16 gene are equal.

The patient died of the widespread metastases two months after surgery, autopsy was not performed.

## Conclusions

On review of the literature, only seven cases of renal mesenchymal chondrosarcoma have been reported**.** Here we first describe an interesting case of renal mesenchymal chondrosarcoma with a synchronous ureter implant, which was intimately mixed or in collision with an urothelial carcinoma. The biphasic growth pattern of highly cellular undifferentiated tumor cells, with islands of differentiated cartilage is rather characteristic of mesenchymal chondrosarcoma [8]. Such a distinctive morphology, with the absence of osteoid formation set mesenchymal chondrosarcoma apart from small cell osteosarcoma, Ewing’s sarcoma, dedifferentiated chondrosarcoma, and hemangiopericytoma.

It is important to note that the absence of epithelial component and of an underlying urothelial carcinoma from an extensively sampled tumor, is an indication of pure mesenchymal chondrosarcoma arising from the kidney. The lack of immunoreactivity for epithelial markers, suck as cytokeratin AE1/E3, EMA and E-cadherin lends further support to it. Moreover, the tumor located in the renal parenchyma and distant inferior ureter, not in the calyx and pelvis, the relatively normal collecting system and urothelium that separates the renal tumor and the distal ureteric mesenchymal chondrosarcoma is keeping with the label of tumor implant.

As for the lower ureteric tumor, the transition between the large bulk of mesenchymal chondrosarcoma and the relatively small urothelial carcinoma was abrupt. Moreover, there was no apparent transition between the carcinoma and the ureteric mucosa or epithelium, which is free of dysplasia or neoplastic field changes. Such an observation support a collision of two separate tumors in the lower ureter. There were some reports on a collision of separate tumors in the same location of the urinary system [9,10]. While only vascular invasion is observed with mesenchymal chondrosarcoma, and no observed with urothelial carcinoma, such a pattern of metastasis does not necessarily imply that the two malignancies are biologically distinct.

From the view of histogenesis, some groups have proposed that a cell could generate a precursor with greater potential than the cell from which it derives, or transdifferentiation, that allows cell lineage switch. Several such multipotential malignant sarcoma have been reported, for example osteosarcoma and Ewing’s sarcoma with epithelial differentiation [11]. The primary tumor cell in mesenchymal chondrosarcoma represents a very primitive mesenchymal cell type. Daniel Rubio clearly demonstrated a novel mesenchymal-epithelial transition (MET) associated epithelial tumorigenesis [12]. And a work showing MET in vitro using a chondrosarcoma tumor cell line has been reported. Fitzgerald MP reported Chondrocytes are mesenchymally derived cells that reportedly acquire some epithelial characteristics, and there is an epigenetic switch associated with an MET-like phenomenon that accompanies chondrosarcoma progression [13]. These observations support the hypothesis that not all carcinomas are derived from epithelial cells. In our present case, both the urothelial carcinoma and the mesenchymal chondrosarcoma all exhibited polysomy of chromosome 3, 7 and 17, the difference was significant (p < 0.05, chi-square test, SPSS 13.0), and loss of p16 gene by fluorescence in situ hybridization (FISH). The development of urothelial carcinoma had been linked to chromosomal instability, especialy chromosome 3,7and 17 and partial or complete loss of chromosome 9 ( p16 locus) [14] . It is thus conceivable that the development of urothelial carcinoma may be triggered or induced by chondrosarcoma, especially since both shared the same clonal origin.

The cytogenetic and molecular mechanism on renal mesenchymal chondrosarcoma is indefinite. Lee AF showed that, in contrast to Ewing sarcoma, small cell osteosarcoma and mesenchymal chondrosarcoma lack FLI-1(Friend leukemia virusintegration-1) immunoreactivity. FLI-1 is therefore useful in the differential diagnosis of small round blue cell tumors of the bone [15]. Wang L described the novel HEY1-NCOA2() fusion appears to be the defining and diagnostic gene fusion in mesenchymal chondrosarcomas [16]. Nevertheless, the progression mechanism of renal mesenchymal chondrosarcoma remains to be developed investigated.

The prognosis for mesenchymal chondrosarcoma arising from bone and soft tissue is dismal. Of the seven cases of primary renal mesenchymal chondrosarcoma reported in the English literature, four cases developed local recurrences or metastases at an interval of 1 month to 2.5 years after surgical intervention. In the current case , whether the ureteric tumor represented a direct intraluminal seeding and ‘implant’ ,or a second primary arising from a background with field change remains speculative, but given its highly aggressive nature, it would be tempting to postulate that this highly aggressive tumor shows a propensity for intra-calyxceal spread. The autopsy was not performed, however vascular mesenchymal chondrosarcoma metastasis detected may implicate mesenchymal chondrosarcoma, rather than carcinoma as the lethal metastasis.

## Consent

Written informed consent was obtained from the next of kin of the patient for publication of this Case Report and any accompanying images. A copy of the written consent is available for review by the Editor-in-Chief of this journal.

## Abbreviations

MRI: Magnetic resonance imaging; Sox-9: Sex determining region Y-box 9; FISH: Fluorescence in situ hybridization; FLI-1: Friend leukemia virusintegration-1; MET: Mesenchymalto epithelial transition.

## Competing interests

The authors have no competing interests.

## Authors’ contributions

HX carried out the molecular genetic studies and participated in draft the manuscript. MMS carried out the immunoassays, participated in the design of the study, participated in draft the manuscript and performed the figures and statistical analysis. HLS participated in its design and clinical data collection, helped in statistical analysis. SML coordination and helped to draft the manuscript. All authors read and approved the final manuscript.
